# Overpressure at the Macondo Well and its impact on the Deepwater Horizon blowout

**DOI:** 10.1038/s41598-019-42496-0

**Published:** 2019-05-07

**Authors:** F. William M. Pinkston, Peter B. Flemings

**Affiliations:** 1 Institute for Geophysics, Jackson School of Geosciences, University of Texas at Austin, Austin, TX 78712 USA; 2 Department of Geological Sciences, Jackson School of Geosciences, University of Texas at Austin, Austin, TX 78712 USA

**Keywords:** Geology, Economic geology

## Abstract

At the Macondo well, the overpressure (fluid pressure greater than hydrostatic) in the main reservoir is nearly identical to that within a stratigraphically equivalent sandstone at the Galapagos development 21 miles (34 km) to the south; we interpret that the reservoirs share a permeable, laterally extensive, and hydraulically connected aquifer. At Macondo, pore pressure approximately parallels the overburden stress to a depth of 17,640 ft (5,377 m) subsea and thereafter decreases abruptly by 1,200 psi (8.3 MPa) over 370 ft (113 m) as the main sandstone reservoir is approached. In contrast, at Galapagos, pore pressure increases with the overburden stress for the entire well depth. The pore pressure regression at Macondo was responsible for a reduction in the least principal stress. This, in combination with the extreme pore pressures within overlying strata, drastically narrowed the range of safe operational borehole pressures. These geologic phenomena produced challenging conditions for drilling, prevented successful temporary abandonment of the well, and contributed to the well’s failure.

## Introduction

**Figure 1:**
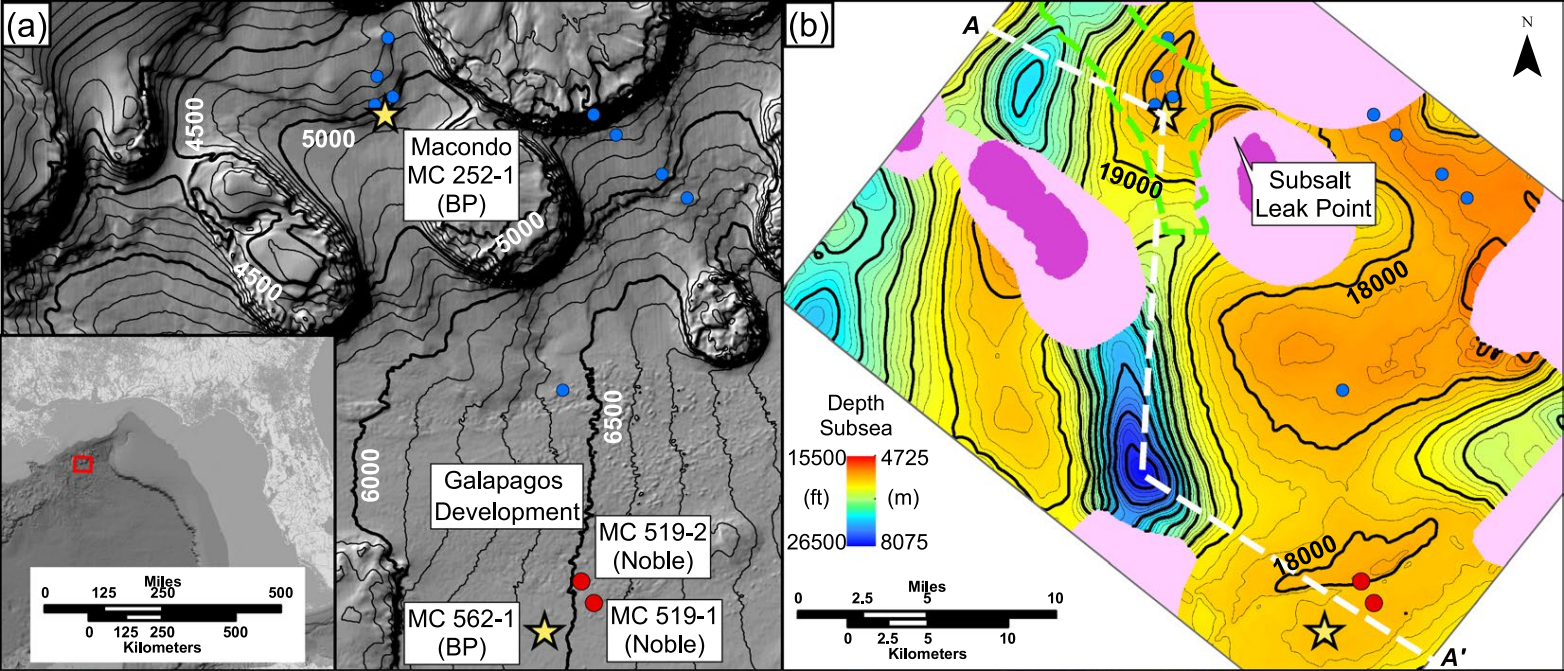
The Macondo well, 252-1, is located 133 miles (214 km) SE of New Orleans in 4,992 ft (1,522 m) of water. Figure 1a,b are collocated and at the same scale. (**a**) Bathymetry map of the study location. Contour interval = 100 ft (30 m). Symbols record bottom-hole locations of wells that penetrate the M56. The Macondo and 562-1 wells are analyzed in this study (Figs 2, 3) The Noble wells (red dots) are used to constrain the aquifer pressure at 562-1. Blue dots locate wells that penetrated the M56 post-blowout. (**b**) The true vertical depth subsea of the M56 interpreted from 3-D seismic data. Contour interval = 250 ft (76 m). Location of the cross-section shown in Fig. 4 is annotated *A*-*A*′ with a white dashed line. The green dashed line denotes the M56 reservoir shape from BP’s exploration plan^15^, but is artificially truncated N-S. The structural map of the M56 reservoir is interpreted from a 3-D seismic volume that is zero-phase, narrow-azimuth, tilted transversely isotropic, and pre-stack reverse-time-migrated in depth. Dark pink indicates truncation of the M56 by salt stocks. The M56 steeply shallows against SE diapir forming a potential fluid leak point. The narrow-azimuth survey does not image bedding well beneath salt (light pink).

On April 20, 2010, the Deepwater Horizon blowout of the Macondo well began in Mississippi Canyon block 252, deepwater Gulf of Mexico (Fig. 1). Eleven people died as a result of the Deepwater Horizon explosion, and over the next three months, an estimated 4 million barrels of oil leaked into the Gulf of Mexico^1^. This human and environmental catastrophe brought to the fore of public consciousness the extraordinary complexity and risk of finding and producing hydrocarbons in the deep ocean. For the first time, the media spotlight focused on the incredible pressures encountered in the search for deepwater hydrocarbons. There has been detailed inquiry into the design and engineering failures that resulted in the blowout^1–6^. However, there has been relatively little public examination of the observations, mechanisms, and implications of the state of pressure and stress in the Macondo well.

We characterize pore pressure and stress within mudstones and sandstones at the Macondo well. We then correlate the sandstone reservoir that was the source of the blowout over an area of 500 mi^2^, and we document that the overpressure within this sandstone 21 miles (34 km) to the southwest (Fig. 1) is within 1.5% of that at Macondo. We interpret that the main reservoir, the M56, is part of a larger hydraulically connected aquifer and present a model to describe the large pore pressure regression present at Macondo. Finally, we summarize how the Macondo pore pressure profile ultimately led to decisions that contributed to the well failure. Our analysis is based on publically available well data archived by the Bureau of Ocean Energy Management (BOEM) and a 3-D seismic volume of Mississippi Canyon. We also gained insights through analysis of documents used during legal proceedings related to the Macondo well^3,6–10^.

## Macondo Pore Pressure Profile

**Figure 2:**
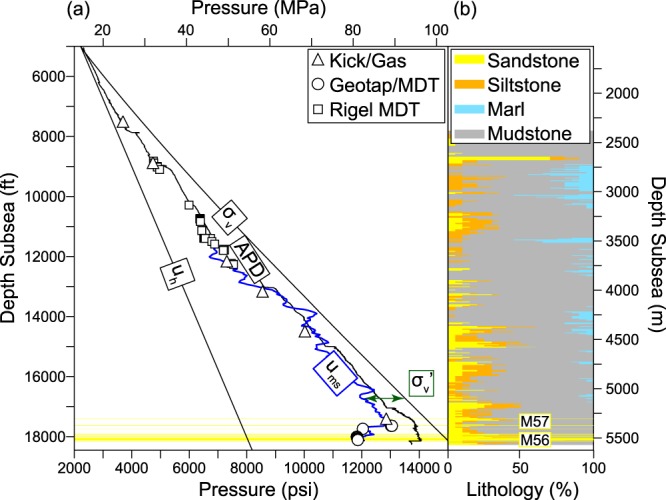
(**a**) Pressure and stress vs. depth beneath sea surface (subsea) from the seafloor to the base of the well. The hydrostatic pressure, *u*_*h*_, assumes a constant fluid density of 1.024 g/cm^3^ (seawater) from the sea surface. The overburden stress, *σ*_*v*_, is calculated by integrating the density of the sediment below the seafloor (see Methods). Direct measurements of pore pressure are shown with symbols (triangles, squares, circles; see Methods for discussion). The mudstone pressure, *u*_*ms*_, interpreted from the (sonic) velocity log is shown with the blue line and the annular pressure-while-drilling (APD) measured near the drill bit is shown with the black line. The depths associated with the M57 and M56 sandstones are highlighted in yellow. (**b**) Lithology penetrated by the Macondo well based on cuttings.

Pore pressures, *u*, in most sedimentary basins are bound below by the hydrostatic pressure, *u*_*h*_, and above the overburden stress, *σ*_*v*_ (see Table 1 for nomenclature). The overpressure, u*, is the pressure above the hydrostatic pressure (u* = *u* − *u*_*h*_). The difference between the overburden stress and the pore pressure is the vertical effective stress (${\sigma ^{\prime} }_{v}={\sigma }_{v}-u$) (Fig. 2a, green). The Macondo pore pressure profile (Fig. 2a) has two basic characteristics. First, from near the seafloor to 17,640 ft (5,377 m), pore pressures approximately parallel the overburden stress and the effective stress is approximately constant. A kick (borehole inflow) documents shallow overpressure at 7,500 ft (2,300 m) (Fig. 2a, black triangle); this is common in deepwater Gulf of Mexico^11^. Second, pore pressure drops as the main reservoir target, the M56 sandstone, is approached. From 17,640 ft (5,377 m) to the base of the well, a pore pressure regression of 1,200 psi (8.3 MPa) is recorded over 370 ft (113 m) between two sandstone packages, the M57 and M56. Most of the pore pressure drop occurs over a vertical distance of just 100 ft (30 m). From the bottom of the M57 at 17,640 ft (5,377 m) to the top of the M56 at 17,740 ft (5,407 m), u falls from 13,050 to 12,050 psi (89.9 to 83.1 MPa).

**Table 1:** Nomenclature. M = Mass, L = Length, t = Time.

Symbol	Name	Dimensions
z_SS_	depth subsea	L^1^
σ_v_′	vertical effective stress	M^1^L^−1^T^−2^
σ_v_	total vertical stress	M^1^L^−1^T^−2^
u	pore pressure	M^1^L^−1^T^−2^
u_ms_	mudstone pore pressure	M^1^L^−1^T^−2^
u*	excess pressure	M^1^L^−1^T^−2^
u_a_*	aquifer excess pressure	M^1^L^−1^T^−2^
u_h_	hydrostatic pore pressure	M^1^L^−1^T^−2^
ϕ	Porosity	—
ϕ_0_	reference porosity	—
ϕ_m_	clay-bound water porosity	—
ρ	bulk density	M^1^L^−3^
ρ_pw_	pore-water density	M^1^L^−3^
g	acceleration of gravity	L^1^T^−2^
FIT	formation integrity test pressure	M^1^L^−1^T^−2^
MDT	modular fm. dynamics tester pressure	M^1^L^−1^T^−2^
v	sonic velocity	L^−1^T
v_ma_	matrix velocity	L^−1^T
T	Temperature	degrees
B	empirical constant^39^	M^−1^L^1^T^2^
x	acoustic formation factor^42^	—
EMW	equivalent mud weight, pounds per gallon (ppg)	M^1^L^−3^

The symbols in Fig. 2a delineate pore pressures measured directly within relatively permeable layers (Fig. 2b, typically sandstones and siltstones); in addition, we estimate the pressure within mudstones, *u*_*ms*_, (Fig. 2a, blue line) from the velocity measured during logging. Our approach to estimating u_ms_ stems from the observation that rock compaction is a function of effective stress^12,13^ and our approach is described in the Methods section. The mudstone pore pressure profile is quite similar to the measured sandstone pressures. From 11,650 to 17,640 ft (3,551 to 5,377 m), u_ms_ increases subparallel to the overburden. Below 17,640 ft (5,377 m), the mudstone pore pressure estimate fully captures the magnitude of the pore pressure regression measured in the M56 reservoir.

## Seismic Interpretation and Stratigraphic Correlation

We map the spatial distribution of the top of the M56 across a 20 by 29 mile (32 by 47 km) area using a 3-D seismic volume (Fig. 1b). We tie the top of the M56 reservoir from projected log data to a reflection in the seismic data, and then track this event across the seismic volume. The top M56 surface ranges from 15,500 to 26,500 ft (4,700 to 8,100 m) resulting in over 11,000 ft (3,350 m) of relief within our study area (Fig. 1b). The map shows one structural high to the north penetrated by the Macondo well, and a second to the south targeted by the Galapagos development. Our mapped surface closely correlates with BP’s independent analysis of the depth of the M56.

The sandstone itself could not be resolved with these seismic data. However, significant sandstones correlate with the M56 surface at every well penetration shown in Fig. 1b. In this region, the transport of sand by turbidity flows in the Middle Miocene was NW to SE^14^. The geologic model from BP’s exploration plan defines the M56 reservoir as an amalgamated, low-relief channel-levee complex that trends NW-SE and has an average thickness of 25–43 ft (7–13 m)^15^. Modern analogs of elongate, continuous, sand-prone channel-levee complexes exceed 30 miles^16^. This characterization of the M56 sandstone (Fig. 1b, dashed green line) is consistent with subsequent reservoir simulation that supports a long narrow aquifer^17^.

## Aquifer Pressure

We compare the aquifer pressure at Macondo with the aquifer pressure at the Galapagos development (Fig. 1). The aquifer pressure is the water-phase pressure in the sandstone and it removes the effect of hydrocarbon buoyancy^18^. The aquifer overpressure, *u*_a_*, is a single number that is independent of depth within a permeable hydraulically connected sandstone^19,20^. At Macondo, we calculate *u*_a_* to equal 3,386 psi (23.35 MPa) whereas at the Galapagos wells, *u*_a_* is 3,433 psi (23.67 MPa) (see Methods). The difference in *u*_a_* between the Macondo and Galapagos locations is 47 psi (0.32 MPa) which is less than 1.5% of the total pressure. We interpret that the nearly identical aquifer pressure records hydraulic connectivity through a shared aquifer.

## Pressure and Stress Profile through the M56 at Macondo and Galapagos

**Figure 3:**
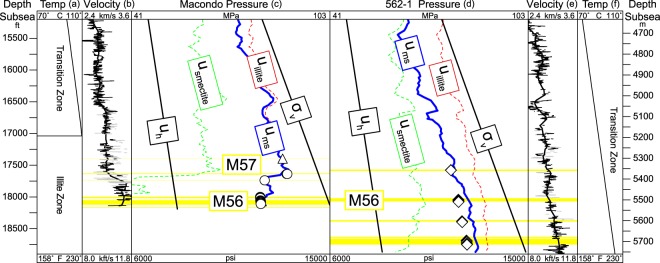
Temperature, mudstone velocity, and pressure vs. depth at the Macondo and 562-1 wells through the M56 reservoir. The modeled mudstone pressure, u_ms_, (blue line) falls abruptly at Macondo (**c**) whereas the mudstone pressure increases continually at 562-1 (**d**). The green and red lines represent the modeled pore pressure for smectitic (green) and illitic (red) mudstone model endmembers as described in the Methods section. Open symbols record pressures in the yellow sandstone intervals. The temperature at the level of the M56 reservoir is 20 °C greater at Macondo than at 562-1 (**a** vs. **f**) (see Methods). The mudstone velocity increases at the M56 level at Macondo whereas it rises continuously at 562-1 (**b** vs. **e**). To show mudstone velocity, the compressional sonic logs (black) were applied a gamma ray cutoff, despike (gray), and a moving average (thick black line).

We compare the pore pressure and velocity profiles across the M56 reservoir at both Macondo and 562-1, one of the Galapagos wells (Fig. 3). The depth below sea surface of the M56 and its pore pressure are essentially identical at the two locations. At 562-1, the pore pressures above and below the M56 (Fig. 3d, symbols) record a continuous and gradual increase in pore pressure with depth subparallel to the overburden. In contrast, the pore pressure at Macondo (Fig. 3c) is much lower within the M56 than above it. The mudstone velocities at both wells (Fig. 3b,e, black lines) increase where the sandstone pressures decrease, reflecting increased compaction due to increased effective stress. At Macondo, there is a sharp increase in mudstone velocity across the M56 interval. The average mudstone velocity (Fig. 3b, thick black line) is 9,500 ft/s (2,900 m/s) across the M57 interval (17,250–17,640 ft or 5,258–5,377 m), but average mudstone velocity increases to 11,000 ft/s (3,350 m/s) across the M56 interval (17,640–18,250 ft or 5,377–5563 m). Although not shown, resistivity and density also increase in this interval, reflecting the increased compaction. In contrast, at 562-1, the velocities show a continuous and gradual increase with depth (Fig. 3e, thick black line). Likewise, our mudstone pressure estimation (Fig. 3d, blue line) is nearly continuous across the M56 at 562-1 in contrast to the pore pressure regression at Macondo.

## Basin Hydrodynamics

**Figure 4:**
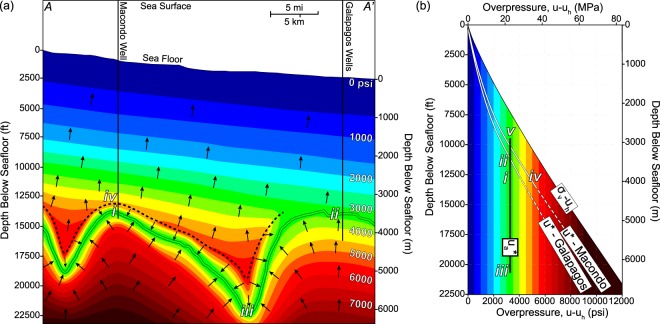
(**a**) Interpreted overpressure cross section *A*-*A*′ (located in Fig. 1). Cooler colors indicate lower overpressure and warmer colors indicate higher overpressure. Arrows are normal to overpressure contours and record the flow direction of pore water within mudstone. The vertical overpressure gradient within the mudstone (contour spacing) decreases from *A* to *A*′ based on observations at the Macondo and Galapagos wells. The black dashed line approximates the flow divide: pore water flows upward above this line and downward below it. The vertical axis shows depth increasing relative to the seafloor. (**b**) Overpressure vs. depth below seafloor. White lines approximate mudstone pore pressures at each well location and become dashed below well control. The vertical black line records a constant overpressure of 3,400 psi (23.4 MPa), approximately what is encountered at Macondo (*i*) and Galapagos (*ii*) in the M56 sand; its top (*v*) and base (*iii*) record the shallowest and deepest mapped location of the M56 sand, respectively. Key locations (*i–v)*: (*i*) M56: Macondo (*ii*) M56: Galapagos (*iii*) M56: deepest mapped depth below seafloor (*iv*) Top of Macondo pore pressure regression (contour reversal) (*v*) Potential leak-off point subsalt (Fig. 1b) where aquifer overpressure converges with fracture pressure (not shown on cross section). Overpressure calculations use a hydrostatic gradient of 0.465 psi/ft, which is based on an aquifer pore-water density of 1.073 g/cm^3^.

We integrate the observations at the Galapagos and Macondo wells with the map of the M56 surface to present a conceptual model of overpressure across the region (Fig. 4a). We have documented nearly constant aquifer overpressure, u_a_*, in the M56 at both locations and we assume u_a_* remains constant between them (Fig. 4, green). From the seafloor down, the mudstone overpressure increases linearly, subparallel with the lithostatic stress as is observed at both Macondo and Galapagos (Figs 2 and 3). At Galapagos, the mudstone pressure is approximately equal to the sandstone pressure at the M56 (Fig. 3d). In contrast, to capture the pore pressure regression at Macondo, there is a reversal in the mudstone pressure trend as the M56 is approached (Fig. 3c); this results in a return to cooler colors (Fig. 4). Beneath the M56, mudstone overpressure again increases. In the conceptual model, contours are connected between wells by assuming a linear decrease in the mudstone overpressure gradient from *A* to *A*′. Contouring adjacent to the M56 assumes the pore pressure regression, if present, is approximately the same distance from the M56 as is observed at Macondo (Fig. 4a, dashed black line).

This overpressure is also expressed in a plot of overpressure vs. depth below seafloor (Fig. 4b). In this view, the constant overpressure of the reservoir at the depths mapped is illustrated with a vertical solid black line. The overpressure in the bounding mudstone away from the reservoir is shown with white lines that represent both Macondo and Galapagos. The M56 pressure is lower than the bounding mudstone pressure at depths below seafloor greater than present at Galapagos (11,650 ft or 3,550 m).

The overpressure cross-section is a fluid potential map: water flows orthogonally to the overpressure contours within material of isotropic permeability. Flow within the mudstone is illustrated by black arrows. In areas where there is a pore pressure regression (Fig. 4a, area between the dashed line and the M56), flow is focused toward the M56. Elsewhere flow is upward: pore pressure gradually dissipates as fluids flow to the seafloor. We interpret that the Galapagos and Macondo reservoirs are hydraulically connected because they have nearly identical aquifer overpressures. In fact, the aquifer pressure at Galapagos is interpreted to be 47 psi (0.32 MPa) greater than at Macondo. In a 2-D view, this implies flow from Galapagos towards Macondo. Although the pressure difference is small, it can drive a lateral flow rate of 200 mm/year given the 300 mD permeability that is estimated for these sandstones.

It is well recognized that in many basins, regionally connected high-permeability aquifers at a nearly constant overpressure are encased in low-permeability overpressured mudstone such as is illustrated here in the M56^21–24^. A key question is, what controls the aquifer overpressure in these systems? One common interpretation is that there is a leak point where the aquifer pressure equals the least principal stress. At this leak point, the pore pressure bleeds off through fractures and the aquifer pressure is fixed to the least principal stress^19,20^. We mapped the M56 reservoir up to 9,500 ft (2,900 m) below the seafloor against the salt diapir 3 miles SE of Macondo (Fig. 1b). At this depth, the aquifer pressure converges to within 1000 psi (6.9 MPa) of the overburden stress (Fig. 4b. ‘*v*’): we interpret that the leak point is at or near this location.

## Implications of the Pore Pressure Regression

**Figure 5:**
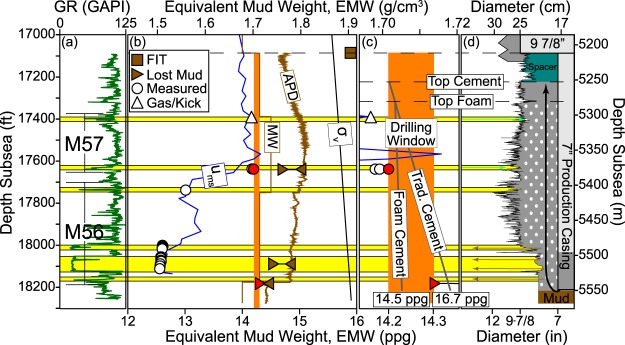
(**a**) Gamma ray log vs. depth with M56 and M57 sand packages defined. (**b**) Pressure and stress gradient vs. depth expressed as an equivalent mud weight (EMW, the average fluid density from the drill floor necessary to reproduce the downhole pressure, see Methods). Lost mud events record the lower and upper bounds of the fracture pressure (brown triangles, see Methods); the formation integrity test (FIT, brown square) records a lower bound of the fracture pressure. The APD is the annular pressure while drilling as recorded on the drill string. The MW (brown line) records the static pressure from drilling mud weight measured at surface conditions. To prevent influx of M57 pore fluids (**c**, green arrows), the static borehole pressure had to be kept above 14.20 ppg (1.702 g/cm^3^) EMW (red circle). However, to avoid fracturing the M56 (**c**, brown arrows), the dynamic pressure had to be kept below 14.3-14.4 ppg (1.714–1.726 g/cm^3^) EMW (red triangle). The zone in orange shows the range of pressures that had to be maintained (the drilling window). (**c**) Pressure and stress gradient vs. depth during temporary abandonment. The two gray lines represent the static pressure that would be induced by a foamed cement (left, 14.5 ppg or 1.738 g/cm^3^) vs. a traditional cement (right, 16.74 ppg or 2.006 g/cm^3^). (**d**) Radial wellbore cross-section with planned casing and cement placement. Caliper measurements record borehole shape. Cement is pumped through the bottom of the casing and up the annulus. White circles differentiate the foamed tail cement from the traditional unfoamed lead and shoe cement pumped before and after. Arrows indicate flow direction if the exposed borehole pressure deviates from the operating window (green, hydrocarbon kick; brown, mud loss).

The pore pressure regression hindered the drilling and temporary abandonment of the Macondo well. To illustrate this, we express the downhole pressures and stresses with an equivalent mud weight (EMW) or density plot (see Methods section) (Fig. 5a,b). Within the exposed borehole, a single mud weight is used to maintain the borehole pressure (1) below the fracture pressure to avoid the loss of drilling fluid (mud) through fractures into the formation and (2) above the pore pressure to prevent flow from the formation into the borehole. The difference between the equivalent mud weight necessary to cause fractures anywhere in the exposed borehole and the EMW that equals the formation pressure anywhere in the exposed borehole is the drilling window. During operations in the deepest well segment at Macondo, the formation was exposed below the base of the 9 7/8″ liner (Fig. 5d). Along this segment, the drilling window was extremely narrow (Fig. 5b,c, orange rectangle): the left bound of this window is constrained by the pore pressure in the M57 of 14.20 ppg (1.702 g/cm^3^) EMW (Fig. 5b,c, red circle) and the right bound is constrained by the fracture pressure (the least principal stress) within the M56 sand (14.3–14.4 ppg (1.714–1.726 g/cm^3^) EMW) (Fig. 5b,c, red triangle).

This narrow drilling window created challenging drilling conditions. Gas flowed into the well from the M57 (Fig. 5b,c, open triangle), indicating that borehole pressures had dropped below the pore pressure. Furthermore, on three occasions, mud was lost into the formation (Fig. 5b, brown triangles), indicating that borehole pressures had exceeded the fracture pressure. In fact, these events constrain the drilling window. The two mud-loss events into the M56 document a lower fracture pressure within this interval than in the upper half of the well segment (Fig. 5b, brown square and uppermost triangle). This drop in fracture pressure (least principal stress) is most likely a result of the reduced pore pressure, but could also be due to different mechanical properties in sands relative to mudstones^25^. The lack of sufficient drilling window meant that BP was forced to terminate drilling without fulfilling all of its objectives, which included drilling to 19,560 ft (5,962 m).

The narrow drilling window impacted the approach used to cement the production casing in place. To maintain the pressures along the cement column within the drilling window (Fig. 5c, gray lines within orange rectangle), BP and Halliburton used 16.74 ppg (2.006 g/cm^3^) cement foamed with nitrogen to reduce its downhole density to 14.5 ppg (1.738 g/cm^3^) to keep dynamic borehole pressures below 14.583 ppg EMW (1.747 g/cm^3^)^26,27^. The particular foam cement mixture was shown to be unstable during testing prior to and after the blowout^28,29^. At trial, it was accepted by both parties that this cement failed, and it was BP’s position that failure of the cement was the primary cause of the failure to seal the well^4,29,30^.

The Macondo well penetrates a complicated hydrogeologic system. A sedimentary section of near lithostatic pressures overlies the lower pressured M56 sand, the exploration target (Fig. 2). The M56 is a regional aquifer whose pressure is likely controlled by a leak point located where the M56 shallows and its pressure converges on the least principal stress (Figs 1, 4b ‘*v*’). To drill and produce this hydrocarbon target required a delicate balance to keep the borehole pressure above the pore pressure present and below the fracture pressure. The technical challenges associated with drilling and cementing this complicated hydrodynamic system contributed to the ultimate blowout of the Macondo well.

## Methods

### Macondo Pore Pressure and Stress Profile

The overburden stress is calculated by integrating the weight of the water column and the weight of the overlying sediment. We combine density log data from nearby wells in portions of the Macondo well where no density data were acquired. Logs are corrected to account for borehole washout and for the presence of hydrocarbons. Where no density data are available, a velocity-to-density transform is used^31^. If neither density nor velocity data are present, an exponential interpolation between density above and below the interval is used^12^.

Industry routinely measures pore pressure and takes fluid samples from relatively permeable formations with wireline tools (e.g. Modular Formation Dynamics Tester^TM^, MDT) and directly from the drill string (Geotap^TM^). At the Macondo well, BP recorded 21 pressures in four sandstones at the base of the well between 17,600 and 18,150 ft (5,364 and 5,532 m) (Fig. 2a, circles). 70 MDT pressures were recorded in nine sandstones between 8,900 and 12,500 ft (2,700 and 3,800 m) (Fig. 2a, squares) at the Texaco 252-1 well, located 1.27 miles (2.04 km) SW of the Macondo well. These MDT measurements are corrected to the Macondo well location assuming continuous stratigraphy parallel to the seafloor^32^.

We also constrain pore pressure from fluid influxes into the borehole (kicks) and elevated gas levels detected in the incoming drilling mud. Kicks and high gas occur when pore pressure exceeds hydraulic pressure from the drilling fluid in the exposed borehole. Six such events occurred during drilling operations (Figs 2, 3 and 5, open triangles). Using drilling information prior-to, during, and after an event, we estimate the location and pore pressure.

Drilling information includes the location of sandstones, length of exposed borehole, gas content of the incoming mud, surface mud weight, equivalent static density, equivalent circulating density, and shut-in drill pipe pressure. The equivalent mud weight is another way of expressing pressure using the average density of the drilling fluid from the drill floor to a location in the borehole. The equivalent static density is the downhole pressure expressed as an equivalent mud weight when the mud pumps are off and thus, there is no circulation. The equivalent circulating density is the downhole pressure expressed as equivalent mud weight as while the drilling fluids circulate. The circulating density is greater than the equivalent static density because of friction caused by fluid circulation.

The fracture pressure is the borehole pressure necessary to hydraulically fracture the formation. It is commonly close to the regional least principal stress but can be affected by stress perturbations due to the borehole geometry and the cohesive strength of the rock. The fracture pressure is constrained at four locations below the 9 7/8″ liner (Fig. 5). The downhole static and dynamic drilling pressures leading up to, during, and after each lost mud event are used to bracket the fracture pressure interpretations (Fig. 5, brown triangles). We define the upper bound of the fracture pressure with the equivalent circulating density when the losses began and the lower bound from the highest static or dynamic pressure at which the well is stable before or after the loss event (see ref.^32^ for detailed explanation). It is generally accepted that the *in-situ* stress of mudstone is higher than that of sandstone^25^, so the loss location is assumed to occur in the sandstone nearest to the bit at the time of the loss event. Fracture pressure is also constrained with the 9 7/8″ formation integrity test, FIT (Fig. 5, brown square). After drilling out of the cemented liner shoe, pressure on the exposed formation was increased to above overburden stress without experiencing fluid loss. This test result provides further evidence that the subsequent losses occurred deeper, in the M56 reservoir interval.

### Mudstone Pore Pressure

Rapid deposition of this low permeability material is the primary source of overpressure in the Gulf of Mexico^33^. It is not practical to directly measure the pressure within these low permeability mudstones. Instead, mudstone pore pressure is commonly estimated from the compaction state (porosity) of the rock, which is typically measured by resistivity, density, or velocity^34,35^. In this approach, a correlation is established between one of these petrophysical proxies and the vertical effective stress, ${\sigma ^{\prime} }_{v}$. Once the correlation is established, then ${\sigma ^{\prime} }_{v}$ is determined given the observed property (e.g. velocity, density, resistivity). Once ${\sigma ^{\prime} }_{v}$ is determined, pore pressure, *u*, is easily determined if the overburden stress, *σ*_*v*_, is known (*u* = *σ*_*v*_ − ${\sigma ^{\prime} }_{v}$).

In deepwater Gulf of Mexico Neogene sediments, pore pressure is not accurately described by a single compaction curve. This is because deeper, hotter, and older mudstones have undergone more compaction than shallower mudstones at the same effective stress. Clay diagenesis is thought to be the primary cause of this behavior and the smectite-to-illite transformation (S/I) is considered the most significant^36–38^. More illitic material has a lower porosity at a given effective stress than a more smectitic material^39,40^. We follow ref.^39^ and assume:

1 $${\rm{\varphi }}-{{\rm{\varphi }}}_{{\rm{m}}}={{\rm{\varphi }}}_{0}{e}^{-B{{\rm{\sigma }}^{\prime} }_{{\rm{v}}}}$$

The left side of Eq. 1 is the total porosity, *ϕ*, less the pore volume that is filled by clay-bound water, *ϕ*_*m*_. The molecular structure of smectite has an easily hydratable interlayer, whereas illite does not^41^; thus the clay-bound water in the illite is less than that in the smectite (*ϕ*_*m*_,_i_ < *ϕ*_*m*_,_s_). The right side of Eq. 1 is a well-established trend for mudstone compaction (e.g. refs^13,35^) and here it describes intergranular porosity loss with effective stress. It is not well known whether *ϕ*_0_ or *B* vary with the degree of the S/I transformation, so we assume that they are constant (ref.^39^)

**Figure 6:**
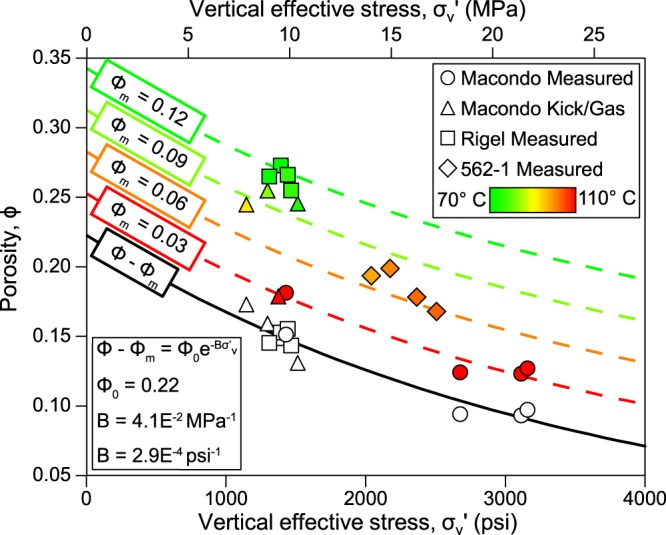
Mudstone porosity vs. effective stress. Color-coded symbols denote *in-situ* temperature for each mudstone porosity-effective stress calibration point. The points are corrected for clay-bound water porosity (open symbols) and then are used to calibrate Eq. 1 (black line). Dashed lines show the porosity-effective stress relationships for different temperatures (color coded) and clay-bound water porosities, *ϕ*_*m*_. Measurements from the M56 (${\sigma ^{\prime} }_{v}$ > 2,500 psi or 17 MPa) are corrected for hydrocarbon buoyancy. Porosity is estimated from velocity (Eq. 2).

We calibrate the model by determining the effective stress within mudstones adjacent to where pressure has been measured in sandstones. We assume that the overpressure, u*, in the mudstone equals u* measured in the nearby sandstone (e.g. ref.^21^), and use the mudstone pressure and overburden to calculate effective stress (*u* = *σ*_*v*_ *−* ${\sigma ^{\prime} }_{v}$). Next, we determine the mudstone porosity at each location from the velocity log after^42^:

2 $${\rm{\varphi }}=1-{(\frac{v}{{v}_{{\rm{ma}}}})}^{1/x}$$

where *v*_*ma*_ is matrix velocity, *v* is the velocity log measurement, and *x* is an empirically derived acoustic formation factor exponent. We assume *x* = 2.19 and *v*_*ma*_ = 14,909 ft/s (4,545 m/s) following precedent for Gulf of Mexico Neogene sediments^21,35,42^. The shallow locations with cooler *in-situ* temperatures have a higher porosity for a given effective stress than the deeper and warmer locations (Fig. 6). This contrast is most apparent at a vertical effective stresses equal to 1,500 psi (10 MPa) where the porosity, *ϕ*, in the shallow section is 9 porosity units greater (Fig. 6, green symbols) than the deeper section (Fig. 6, red symbols). We interpret that the deeper sediments have lost clay-bound water *ϕ*_*m*_ as the smectite in the mudstone was converted to illite with burial.

We assume that porosity loss from clay-bound water release during the S/I transformation is linearly proportional to temperature, and that transformation begins at 70 °C and plateaus at 110 °C. This approximates the main phase of S/I transformation^43–45^ without additional constraints on depositional history and chemical composition^46^. We follow Lahann^39^ and assume *ϕ*_*m*_ = 0.12 for smectitic mudstone and *ϕ*_*m*_ = 0.03 for illitic mudstone. Based on these assumptions, the clay-bound water porosity is:

3 $${{\rm{\varphi }}}_{{\rm{m}}}=(1-\frac{{\rm{T}}-{{\rm{T}}}_{{\rm{s}}}}{{{\rm{T}}}_{{\rm{i}}}-{{\rm{T}}}_{{\rm{s}}}})({{\rm{\varphi }}}_{{\rm{m}},{\rm{s}}})+\frac{{\rm{T}}-{{\rm{T}}}_{{\rm{s}}}}{{{\rm{T}}}_{{\rm{i}}}-{{\rm{T}}}_{{\rm{s}}}}({{\rm{\varphi }}}_{{\rm{m}},{\rm{i}}})$$

where *T* is temperature, and *T*_*s*_ and *T*_*i*_ are the smectite (70 °C) and illite (110 °C) transformation boundary temperatures. We combine Eqs 2 and 3, and solve for *ϕ* *−* *ϕ*_*m*_ for all the *ϕ* vs. ${\sigma ^{\prime} }_{v}$ points in Fig. 6. We then use least-squares regression to constrain Eq. 1 and find *ϕ*_0_ = 0.22 and *B* = 2.9E^−4^ psi^−1^ (Fig. 6, black line).

Given *B* and *ϕ*_0_, Eq. 1 is then used to estimate mudstone pressure along the borehole (Fig. 2a, blue line) with *ϕ*_*m*_ calculated from Eq. 2. To calculate mudstone velocity, we picked mudstones along the borehole at 30–40 ft (9–12 m) intervals and applied a 5-pick moving average to the corresponding compressional sonic log measurements. For each mudstone pick, we calculate *ϕ* from mudstone velocity (Eq. 2) and *ϕ*_*m*_ from temperature (Eq. 3). *ϕ* and *ϕ*_*m*_ are entered into Eq. 1, solving for ${\sigma ^{\prime} }_{v}$ and then *u*.

We apply this method (calibrated at Macondo) to estimate the mudstone pressure at 562-1 (Fig. 3). The close match between the estimated mudstone pressures and the measured sandstone pressures, independent of local calibration, supports the accuracy of our method within this region. Effective stresses at 562-1 are roughly 500-1,300 psi (3–9 MPa) higher than at Macondo (outside of the pressure regression). Mudstone sonic porosities are similar in both wells, but the temperature gradients are different. The Macondo well has an average temperature gradient of 28.4 °C/km versus 26.1 °C/km at 562-1. The lower temperature gradient and deeper water at 562-1 results in M56 temperatures that are nearly 20 °C lower than M56 temperatures at Macondo. The lower temperature indicates that the mudstone at 562-1 is more smectitic than the mudstone at Macondo for a given depth, so the sonic porosities transform to higher ${\sigma }_{v}^{\prime}$ (Fig. 6).

### Aquifer Pressure

**Figure 7:**
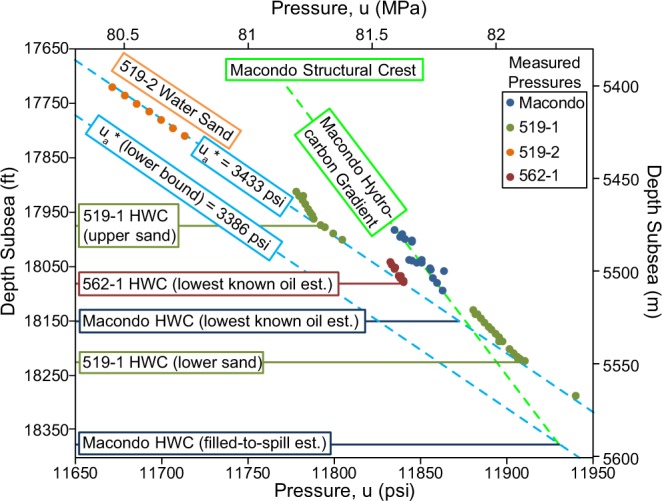
Pressure vs. depth of M56 MDT measurements from four wells. Water-phase pressures for the Macondo and Galapagos structures are shown as blue dashed lines. A green dashed line denotes the M56 hydrocarbon gradient at Macondo. Solid horizontal lines locate observed and estimated hydrocarbon-water contacts.

We determine the M56 aquifer overpressure at the Macondo well to be 3,386 psi (23.35 MPa), but it could be as high as 3,436 psi (23.69 MPa). At the Galapagos development, the M56 aquifer overpressure is tightly constrained to equal 3,433 psi (23.67 MPa). The overpressures are constrained with direct pressure measurements in the M56 sandstones at the Macondo well and three wells at the Galapagos development (Figs 1, 7). These wells are chosen because the pressure measurements were made before production at either location; thus, the measurements are interpreted to record the *in-situ* pressures unaffected by production or the Macondo release (Fig. 1, red circles and yellow stars). Many of the measurements were made within hydrocarbon-bearing sections. To determine the aquifer overpressure in such cases, the buoyant effect of the hydrocarbon column must be removed (e.g. ref.^18^). Specifically, the hydrocarbon pressure is projected down to the hydrocarbon-water contact (HWC) using the MDT-derived hydrocarbon density (Fig. 7). For each well at Macondo and Galapagos, we constrain the HWC, hydrocarbon-phase density, and water-phase density with log, MDT and seismic data. We then calculate aquifer overpressure at Macondo and Galapagos, taking into account pore-water density (*u*_a_* = *u* − *ρ*_*pw*_*gz*_*SS*_).

At Macondo, we interpret that the 4-way closure of the M56 structure (Fig. 1b) was filled to its spill point. We interpret a structural crest at 17,720 ft (5401 m), a saddle at 18,375 (5601 m), and thus a column height of 655 ft (200 m) by depth-correcting BP’s predrill interpretation^15^. BP interpreted that the seismic amplitudes supported this filled-to-spill interpretation for the HWC^15^. We calculate the aquifer overpressure, *u*_a_*, to equal 3,386 psi (23.35 MPa) using a hydrocarbon gradient of 0.24 psi/ft (5.43 MPa/km) and a pore-water gradient of 0.465 psi/ft (10.52 MPa/km). It is possible that the structure was not filled to spill thus the HWC is shallower. LLOG-253-1 (Fig. 1, northernmost blue dot) provides the deepest hydrocarbon-bearing penetration of the M56 in the Macondo structure at 18,150 ft (5,532 m), which yields an upper bound to the aquifer overpressure of 3,436 psi (23.69 MPa)

The three Galapagos development wells (519-1, 519-2, and 562-1) (Fig. 1) constrain the aquifer pressure at this location to a single value (Fig. 7). At 519-1, two vertically stacked sandstone lobes comprise the M56. Each lobe shows a distinct HWC, but both share a *u*_a_* of 3,436 psi (23.69 MPa). 519-2 encountered only water in the M56, which yields *u*_a_* of 3,430 psi (23.65 MPa). We use these 519-2 MDT measurements to estimate the M56 pore water density of 0.465 psi/ft (10.52 MPa/km). 562-1 encountered hydrocarbon in the M56 and did not penetrate a HWC. An aquifer pressure calculation that assumes the HWC is just below the sandstone yields a *u*_a_* of 3,433 psi (23.67 MPa), which is nearly identical to those observed in the 519-1 and 519-2 wells. We use the average, 3,433 psi (23.67 MPa), to describe the aquifer overpressure at the Galapagos development.

### Temperature Profiles

**Figure 8:**
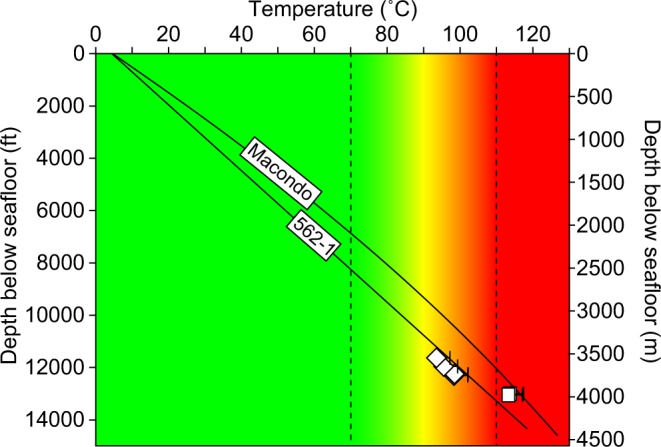
Temperature vs. depth below seafloor at Macondo and 562-1. Open symbols show MDT pore fluid temperature measurements. Error bars projected from the right represent a correction for borehole cooling. BP’s temperature model is used at Macondo; 562-1 temperatures are modeled using a linear projection to the seafloor. Color scheme and dotted lines show the temperature-derived S/I transition zones.

We determined the temperature profiles at Macondo and 562-1 using temperatures recorded during MDT pore fluid sampling (Fig. 8, open symbols). Temperatures between 113.3 and 113.7 °C were recorded at three MDT sample points in the Macondo well between 13,008 and 13,064 ft (3,965 and 3,982 m) below seafloor (Fig. 8, rectangles). At 562-1, four MDT sample points record temperatures between 93.5 and 98.4 °C for depths between 11,633 and 12,316 ft (3,545 and 3,754 m) below seafloor (Fig. 8, diamonds). BP’s temperature model for Macondo (Fig. 8, upper black line)^8^ is 3.8 °C higher than the average of the recorded temperatures in the M56 (Fig. 8, rectangle error bars). We assume this difference reflects a correction for borehole cooling. At Macondo, MDT measurements were acquired three days after drilling was completed, which is comparable to the four day gap at 562-1. Therefore, we apply the same 3.8 °C correction to the measurements at 562-1 (Fig. 8, diamond error bars). Our temperature model for 562-1 assumes a linear decrease from the corrected reservoir measurements to the seafloor (Fig. 8, lower black line). Seafloor water temperatures in deepwater Gulf of Mexico approach 4 °C for the water depths observed at Macondo and 562-1.

## Data Availability

The data that support the findings of this study are available in this thesis^32^ and by request from the corresponding author F.W.M.P. Trial related documents are available at http://www.mdl2179trialdocs.com. The seismic data are not publicly available due to license restrictions.
